# Whole-Transcriptome and -Genome Analysis of Extensively Drug-Resistant Mycobacterium tuberculosis Clinical Isolates Identifies Downregulation of *ethA* as a Mechanism of Ethionamide Resistance

**DOI:** 10.1128/AAC.01461-17

**Published:** 2017-11-22

**Authors:** Lynne de Welzen, Vegard Eldholm, Kashmeel Maharaj, Abigail L. Manson, Ashlee M. Earl, Alexander S. Pym

**Affiliations:** a Africa Health Research Institute (AHRI), School of Laboratory Medicine & Medical Sciences, University of KwaZulu-Natal, KwaZulu-Natal, South Africa; b Infectious Disease Control and Environmental Health, Norwegian Institute of Public Health, Oslo, Norway; c Broad Institute of MIT and Harvard, Cambridge, Massachusetts, USA; d University College London (UCL), London, United Kingdom

**Keywords:** promoter, RNA sequencing, RNA-seq, drug resistance, drug susceptibility testing, flow cytometry, fluorescent reporter, *mymA*, tuberculosis

## Abstract

Genetics-based drug susceptibility testing has improved the diagnosis of drug-resistant tuberculosis but is limited by our lack of knowledge of all resistance mechanisms. Next-generation sequencing has assisted in identifying the principal genetic mechanisms of resistance for many drugs, but a significant proportion of phenotypic drug resistance is unexplained genetically. Few studies have formally compared the transcriptomes of susceptible and resistant Mycobacterium tuberculosis strains. We carried out comparative whole-genome transcriptomics of extensively drug-resistant (XDR) clinical isolates using RNA sequencing (RNA-seq) to find novel transcription-mediated mechanisms of resistance. We identified a promoter mutation (t to c) at position −11 (t−11c) relative to the start codon of *ethA* that reduces the expression of a monooxygenase (EthA) that activates ethionamide. (In this article, nucleotide changes are lowercase and amino acid substitutions are uppercase.) Using a flow cytometry-based reporter assay, we show that the reduced transcription of *ethA* is not due to transcriptional repression by *ethR*. Clinical strains harboring this mutation were resistant to ethionamide. Other *ethA* promoter mutations were identified in a global genomic survey of resistant M. tuberculosis strains. These results demonstrate a new mechanism of ethionamide resistance that can cause high-level resistance when it is combined with other ethionamide resistance-conferring mutations. Our study revealed many other genes which were highly up- or downregulated in XDR strains, including a toxin-antitoxin module (*mazF5 mazE5*) and tRNAs (*leuX* and *thrU*). This suggests that global transcriptional modifications could contribute to resistance or the maintenance of bacterial fitness have also occurred in XDR strains.

## INTRODUCTION

Mycobacterium tuberculosis, the causative agent of tuberculosis (TB), has progressively developed resistance to the most effective first- and second-line antituberculosis drugs (1). Patients infected with extensively drug-resistant (XDR) strains (strains resistant to the fluoroquinolones and aminoglycosides, in addition to rifampin [RIF] and isoniazid, in which resistance to the last two drugs defines multidrug resistance [MDR]) have extremely high rates of mortality, despite the use of long and intensive treatment regimens (2, 3). The ultimate control of drug resistance will require multiple interventions, one of which will be individualized therapy based on rapid comprehensive drug susceptibility testing (DST).

Current molecular genetics-based tests, such as the GeneXpert MTB/RIF and GenoType MTBDRplus assays, have accelerated the clinical detection of known mutations causing RIF and/or isoniazid resistance (4, 5). These and other genetic tests detect only MDR-TB and a limited number of mutations associated with resistance to second-line drugs (6). Whole-genome sequencing (WGS) has the potential to rapidly detect all possible drug resistance-conferring mutations (7). However, recent studies have demonstrated that genotypic DST using WGS lacks sensitivity for the detection of resistance to many second-line drugs, including fluoroquinolones (8–11). Improving the sensitivity of genetic susceptibility testing will be possible only with a more comprehensive understanding of the genetic determinants of drug resistance.

Our current understanding of drug resistance in M. tuberculosis has developed through studying resistant mutants isolated *in vitro* and the accumulation of mutations in resistant clinical isolates (12). These studies have identified various genetic mechanisms of resistance, including target modification, loss of the enzymatic function required to activate prodrugs, and altered drug efflux (13, 14).

In addition to intragenic mutations, there is increasing evidence that alterations to gene transcription are an important mechanism of conferring drug resistance. Promoter mutations which result in the upregulation of *inhA*, which encodes the target for isoniazid, were the first to be described (15). Pyrazinamide (PZA) resistance has been associated with mutations in the regulatory region upstream of *pncA*, the enzyme responsible for activating PZA (16–18). Aminoglycoside cross-resistance in M. tuberculosis can arise due to mutations in the regulatory region of *whiB7* (which encodes a transcriptional activator), which results in increased expression of *eis* (which acetylates and inactivates kanamycin), as well as *tap* (which encodes an efflux pump that extrudes streptomycin) (19). *eis* promoter mutations have also been described. Recently, cross-resistance between clofazimine (CFZ) and bedaquiline (BDQ) was shown to be due to mutations within *Rv0678* (20, 21), a transcriptional repressor, which results in derepression and upregulation of the multisubstrate efflux pump *mmpL5*.

Despite the discovery of these varied transcriptionally driven mechanisms of resistance, there have been few systematic whole-genome transcriptional comparisons of suitably matched susceptible and resistant M. tuberculosis strains, and none to date has used RNA-sequencing (RNA-seq). In this study, we therefore selected phylogenetically closely related susceptible and resistant clinical strains and subjected them to comparative transcriptomics using RNA-seq to identify novel mechanisms of resistance.

## RESULTS

### Comparative transcriptomics.

**FIG 1:**
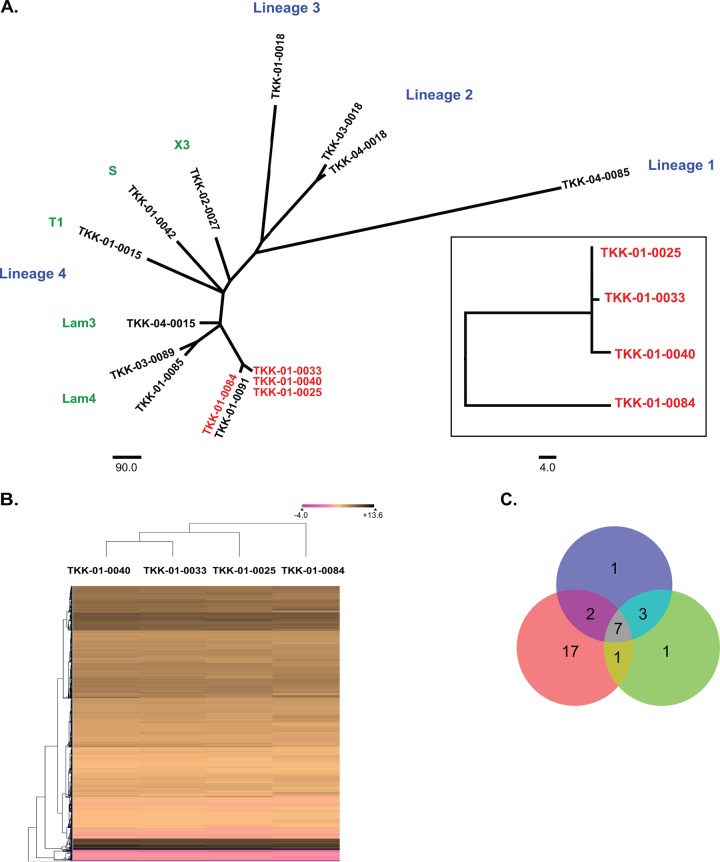
(A) Phylogenetic tree representing the distribution of the 4 strains selected for RNA-seq (shown in red and boxed). (B) Hierarchical gene clustering of the 4 strains selected for RNA-seq on the basis of their relative gene expression shows that the drug-susceptible strain clusters separately from the others. (C) Venn diagram representing the numbers of genes differentially expressed at levels 7-fold or greater relative to their levels of expression by the susceptible control strain. The blue, red, and green circles represent pairwise comparisons with TKK-01-0033, TKK-01-0025, and TKK 01-0040, respectively.

In order to identify novel mechanisms of resistance mediated at the level of transcription, we subjected drug-resistant and drug-susceptible strains of M. tuberculosis to comparative transcriptomics using RNA sequencing (Table 1). We reasoned that strains with highly complex resistance profiles were most likely to have acquired mutations resulting in transcriptional changes. Using a whole-genome-based phylogenetic analysis, we identified 3 XDR clinical isolates from a well-documented outbreak in KwaZulu-Natal, South Africa, and a closely related drug-susceptible strain to act as a control (1). All strains were from the LAM4 branch of lineage 4. In pairwise comparisons, the 3 XDR strains differed from each other by 7 single nucleotide polymorphisms (SNPs) or less (Fig. 1A). The maximum difference between the drug-susceptible strain and an XDR strain was 76 SNPs, of which 6 occurred in known drug resistance-conferring genes.

**TABLE 1:** Strain details, including resistance mutations and RNA sequencing coverage

Strain	Spoligotype	Resistance mutation for each drug^*a*^							RNA-seq coverage (fold)
		INH	RIF	STR	EMB	KAN	ETH	OFL	
TKK-01-0084	LAM4								288.63
TKK-01-0025	LAM4	*inhA* t−8a	*rpoB* L452P	*gidB* L16R	*embB* M306V	*rrs* A1401G	*inhA* t−8a	*gyrA* A90V	214.34
		*katG* S315T	*rpoB* D435G	*gidB* del					
TKK-01-0033	LAM4	*inhA* t−8a	*rpoB* L452P	*gidB* L16R	*embB* M306V	*rrs* A1401G	*inhA* t−8a	*gyrA* A90V	239.13
		*katG* S315T	*rpoB* D435G	*gidB* del					
TKK-01-0040	LAM4	*inhA* t−8a	*rpoB* L452P	*gidB* L16R	*embB* M306V	*rrs* A1401G	*inhA* t−8a	*gyrA* A90V	269.24
		*katG* S315T	*rpoB* D435G	*gidB* del					

a INH, isoniazid; RIF, rifampin; STR, streptomycin; EMB, ethambutol; KAN, kanamycin; ETH, ethionamide; OFL, ofloxacin; del, deletion.

To determine if there were global transcriptional differences between our strains, we first carried out hierarchical clustering of their transcriptional profiles. This separated the expression profiles of the three drug-resistant strains from the expression profile of the susceptible control (Fig. 1B). To identify genes either up- or downregulated in the XDR strains, we performed pairwise comparisons for each resistant strain with the drug-susceptible control. In the resistant strains, up to 40 genes were significantly over- or underexpressed at the 95% confidence level (*P* ≤ 0.05) and up to 10 genes were significantly over- or underexpressed at the 99% level (*P* ≤ 0.01) relative to their expression in the susceptible control (see Table S1 in the supplemental material). Importantly, in all three pairwise comparisons, the *inhA* gene showed a greater than 8-fold upregulation of expression in the resistant strains at the 99% confidence interval. All three resistant strains harbored a t-to-a mutation at position 8 (t−8a) in the promoter region of *fabG1*, which is known to cause the upregulation of *inhA*. The detection of this transcriptional change therefore acted as an internal validation of our approach. Apart from *inhA*, no other genes were significantly upregulated in all three comparisons. Two genes, *fabG1* (also in the *inhA* operon) and *Rv1761c* (a gene of unknown function), had expression levels in two strains (TKK-01-0040 and TKK-01-0033) significantly different from that in the susceptible control.

After defining differential gene expression at the statistically significant levels (95% and 99% confidence intervals), we extended our analysis to all genes that had a high mean fold change in transcript levels (≥7-fold up or down) relative to the susceptible control (Fig. 1C and Table S2). In addition to *fabG1* and *inhA*, we found that 5 other genes fell into this classification: *mazF5*; *mazE5*, encoding a toxin-antitoxin module; two tRNAs (*leuX* and *thrU*); and *ethA. ethA* was of particular interest, as it encodes a monooxygenase required for the activation of the prodrug ethionamide (ETH) (22, 23), a key component of treatment of infections cause by MDR strains. Loss-of-function mutations in *ethA* result in ethionamide resistance (23, 24). Following Benjamini-Hochberg correction, *ethA* was found to be significantly downregulated in one of our pairwise comparisons described above.

### Comparative genome-transcriptome analysis.

**FIG 2:**
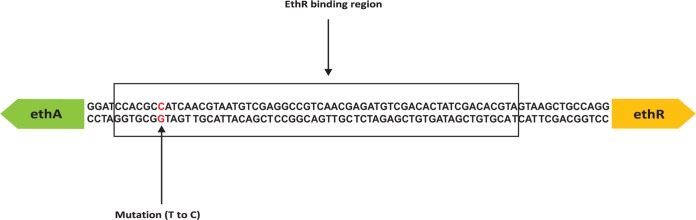
Representation of the intergenic region between *ethA* and *ethR*. The location of the single nucleotide polymorphism (SNP) is found 11 bp upstream of *ethA* and is indicated in red. The *ethR* binding region is indicated by the black box (25).

In order to understand the genetic basis of the transcriptional changes defined by our RNA-seq experiments, we used comparative genomics to identify mutations located in intergenic regions associated with genes that were highly over- or underexpressed in our resistant strains relative to our susceptible control strain. This analysis identified an intergenic region mutation (t to c) at position −11 (t−11c) relative to the start codon of *ethA*. The detected mutation was located within the promoter region of *ethA* as well as within the binding domain of the divergently expressed transcriptional regulator *ethR*, which is known to repress *ethA* (25) (Fig. 2). The location of the mutation suggested that it could lead to the downregulation of EthA by (i) directly reducing *ethA* transcription independently of *ethR* regulation, (ii) increasing *ethR* transcription, leading to the repression of *ethA*, or (iii) affecting the binding of *ethR*, leading to the increased repression of *ethA* transcription.

### Functional characterization of *ethA* and *ethR* promoters.

**FIG 3:**
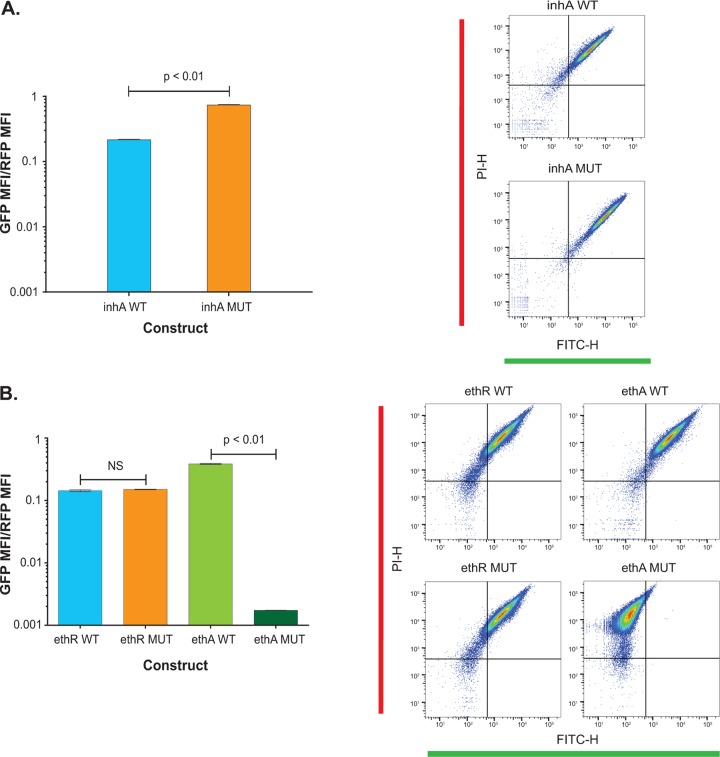
Analysis of promoter activity between the wild-type (WT) and mutant (MUT) constructs. (Left) Ratios of the median fluorescent intensity (MFI) of the green fluorescent protein (GFP) to the MFI of the red fluorescent protein (RFP), as well as statistical differences between the wild-type and mutant constructs for the *inhA* promoter (A) and *ethA* and *ethR* promoters (B). *P* values are indicated on the bar charts. (Right) Single cell counts from flow cytometry. RFP expression is represented on the *y* axis as the PI-H (height) channel, and GFP expression is represented on the *x* axis as the FITC-H (height) channel.

To determine if the t−11c mutation functionally influenced either *ethA* or *ethR* transcription, we used a dual-color fluorescent protein promoter assay. The episomal construct pLDW-DC* has a constitutively expressed red fluorescent protein (RFP), TagRFP, and a promoterless Emerald green fluorescent protein (GFP), in front of which promoters with or without mutations can be cloned. Promoter activity is expressed as the ratio of green to red fluorescence, normalizing for any variability in plasmid number. To validate our approach, we used the *fabG1-inhA* promoter with and without the mutant promoter sequence of *inhA* with a g−17t mutation. The construct harboring the g−17t mutant promoter sequence of *inhA* resulted in a 3.4-fold increase in the ratio of the median fluorescent intensity (MFI) of green fluorescence to the MFI of red fluorescence (the MFI ratio) relative to the MFI ratio for the wild type (Fig. 3A).

We then assayed constructs with the wild-type 250-bp upstream region of *ethA* or *ethR* and 2 matched mutant constructs with either the t−11c mutation (relative to *ethA*) or the corresponding t−65c mutation in the *ethR* construct (Fig. 3B). We observed no significant change in the MFI ratio between the two *ethR* promoter constructs. In contrast, the t−11c mutant promoter resulted in an MFI ratio that was significantly lower than that obtained with the wild-type control. These results suggest that the t−11c intergenic region mutation does not affect the transcription of *ethR* but does diminish the expression of *ethA* to levels that could result in ethionamide resistance.

### *ethA* expression in clinical isolates of M. tuberculosis.

**FIG 4:**
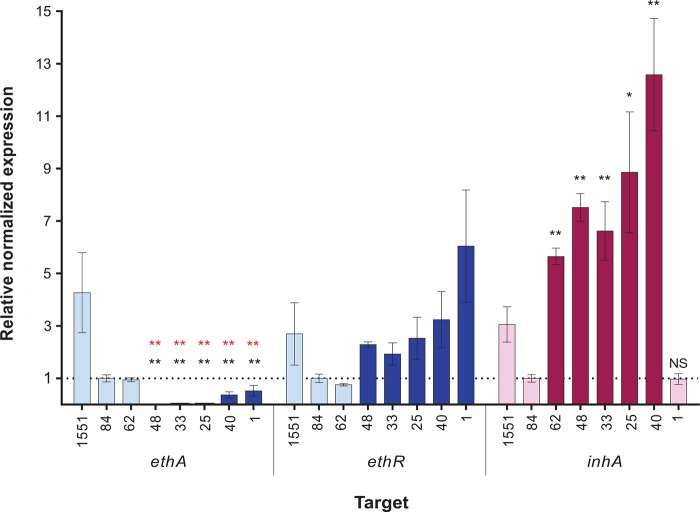
Relative gene expression of *ethA*, *ethR*, or *inhA* in clinical strains of M. tuberculosis (see Table S5 in the supplemental material). Gene expression levels were normalized to the *sigA* expression level for each strain. Relative normalized expression represents the fold change in the normalized expression by each strain compared to that by drug-susceptible clinical strain TKK-01-0084. Light blue bars, strains that do not contain t−11c *ethA* promoter mutations; dark blue bars, strains that have the t−11c *ethA* promoter mutation; light pink bars, strains without *inhA* promoter mutations; dark pink bars, strains with *inhA* promoter mutations. The TKK strain numbers are abbreviated to their last two digits; e.g., 62 represents strain TKK-01-0062. The statistical significance of the relative normalized expression for *ethA* and *inhA* was derived using unpaired *t* tests between each strain and clinical drug-susceptible strain TKK-01-0084 (asterisks in black). In addition, the statistical significance of the relative normalized expression for *ethA* was derived using unpaired *t* tests between each strain and strain TKK-01-0062, which does not harbor a t−11c *ethA* promoter mutation (asterisks in red). 1551 corresponds to the laboratory strain CDC1551, which was excluded from this analysis. **, *P* ≤ 0.01; *, *P* ≤ 0.05; NS, not significant.

To confirm the transcriptional changes identified by RNA-seq in strains harboring the t−11c mutation, we used quantitative reverse transcription-PCR (RT-qPCR) to measure the expression levels of *ethA* in clinical isolates (Table S3). In the 5 strains with an *ethA* t−11c promoter mutation tested, the relative normalized expression levels of the monooxygenase were significantly lower or close to zero compared to those in the control strains. All tested strains with the *inhA* promoter mutations had increased relative normalized levels of expression of *inhA* compared to those in strains without the mutation (Fig. 4).

### *ethA* promoter mutations and ethionamide resistance in clinical isolates.

To determine if *ethA* promoter mutations were associated with clinical resistance, we tested a panel of clinical isolates which, on the basis of genome analyses, harbored putative ethionamide resistance-conferring mutations for quantitative ethionamide susceptibility (Table 2). The panel included three strains that had the t−11c intergenic region mutation but no other mutations previously associated with clinical ethionamide resistance (*inhA* promoter mutations and intragenic region mutations in *ethA*, *ethR*, *ndh*, and *mshA*) (26). Recently, loss-of-function mutations in another M. tuberculosis monooxygenase, *mymA* (*Rv3083*), have been proposed to be an additional resistance mechanism (27). Interestingly, during our selection of strains, we were able to identify a group of isolates with a deletion spanning *mymA* (Fig. S1). Sequence confirmation in five of these strains showed an identical deletion of 2,891 bp, indicating a unique polymorphism event suggestive of clonal expansion. Strains with this mutation were included in our analysis.

**TABLE 2:** ETH MICs for clinical strains^*a*^

Strain	DST	Spoligotype	Putative ETH resistance-conferring mutations								MIC (mg/liter)
			*ethA* promoter	*inhA* promoter	*inhA* intragenic region	*ethA* intragenic region	*ethR* intragenic region	*mymA*	*mshA*	*ndh*	
TKK-01-0001	MDR	KZN	t−11c								20
TKK-01-0035	MDR	KZN	t−11c								10
TKK-01-0075	MDR	KZN	t−11c								5
TKK-01-0025	XDR	KZN	t−11c	t−8a							80
TKK-01-0040	XDR	KZN	t−11c	t−8a							>80
TKK-01-0048	MDR	KZN	t−11c	t−8a							20
TKK-01-0033	XDR	KZN	t−11c	t−8a							80
TKK-02-0001	XDR	Beijing		c−15t	I194V		P94P		A189T		40
TKK-02-0046	Poly (P/N + RIF)	Beijing		c−15t	I194V		P94P		A189T		20
TKK-01-0005	Poly (STR + ETH)	Beijing		c−15t					A189T		80
TKK-01-0062	PXDR	Beijing		g−17t		A381P					20
TKK-01-0032	MDR	S		t−8g						V18A	5
TKK-01-0013	XDR	Beijing				Y276H			A189T		5
TKK-02-0018	MDR	T3				V202L		del			15
TKK-02-0019	PXDR					V202L		del			20
TKK-01-0026	MDR	T3						del			2.5
TKK-02-0069	PXDR							del			5
TKK-01-0081	DS	KZN									2.5
TKK-01-0084	DS	KZN									2.5
TKK-01-0047	DS	Beijing									2.5
TKK-01-0027	DS	Beijing									2.5
H37Rv	DS	NA									2.5
CDC 1551	DS	NA									1.25

a del, deletion; Poly, polydrug-resistant (resistance to one or more drugs but not MDR/XDR); P/N, para-aminosalicylic acid–nicotinamide; RIF, rifampin; STR, streptomycin; ETH, ethionamide; PXDR, preextensively drug resistant (MDR and resistance to either a fluoroquinolone or an injectable); DS, drug susceptible; NA, not applicable.

The ETH MICs for strains that had only the *ethA* t−11c promoter mutation ranged from 5 to 20 mg/liter, showing low-level resistance to ETH (Table 2). However, in combination with the t−8a *inhA* promoter mutation, we observed higher levels of resistance, suggesting that the phenotypic consequences of the two promoter mutations are additive. The two strains with only *mymA* deletions had ETH MICs of 2.5 and 5 mg/liter, which were only marginally elevated relative to the MICs of strains without any ethionamide drug resistance-conferring mutations (1.25 to 2.5 mg/liter).

### Global distribution of *ethA* promoter mutations.

In order to determine how widespread *ethA* promoter mutations are among clinical M. tuberculosis isolates, we exploited a recent genome analysis of globally isolated drug-resistant and -susceptible strains (28). From a total of 5,310 strains, we identified 402 with a mutation within the *ethA-ethR* intergenic region relative to the sequence of H37Rv (Table S4). One hundred thirty-nine of these were the t−11c mutation, and all were identified in South Africa-derived lineage 4 isolates. The most common mutation was a−7g, found in 212 strains, nearly all of which (205 strains) were from Eastern Europe. Eleven other infrequently occurring mutations were identified, but one of these also mapped to the −11 site (t−11g). Using parsimony to define independent mutational events across the phylogeny (29), we found that the a−7g mutation had independently evolved at least 32 times, suggesting that this mutation was under selective pressure and supporting the possibility that it has a role in conferring drug resistance. In contrast, the t−11c mutation was predicted to have evolved only once, which is compatible with the ongoing transmission and clonal expansion of XDR strains from South Africa in which the mutation was found.

## DISCUSSION

The aim of our study was to use a comparative whole-genome transcriptomic approach to identify novel mechanisms of resistance mediated at the transcriptional level. We were able to identify a promoter mutation upstream of *ethA*. We confirmed by our dual-color promoter assay and quantitative RT-PCR that this mutation leads to the reduced transcription of *ethA*, which encodes a monooxygenase that activates the prodrug ethionamide (22, 23). Strains harboring only this t−11c mutation and no other ethionamide resistance-determining genotypes were resistant to ethionamide (MIC range, 5 to 20 mg/liter), indicating that this mutation should be included in genetics-based diagnostic tests. In support of this, a recent genomewide association analysis also reported an association between the t−11c mutation and ethionamide resistance (8).

In our analysis of the global distribution of *ethA-ethR* intergenic region mutations, we identified the t−11c mutation solely in South African strains. This data set, however, consisted of isolates from only 43 countries and notably lacked representation from several regions of the world where TB is epidemic, such as South America. Nonetheless, a previous study using direct sequencing of drug resistance loci detected five different variants in the promoter region of *ethA*, one of which was a t−11c mutation found in an isolate from Peru (30). There were, however, no clinical data available to rule out whether the patient in question had any travel history to South Africa. We therefore cannot confirm whether this mutation is geographically restricted. The a−7g mutation was more dispersed, but the majority of strains harboring this mutation were from Eastern Europe. A previous study reported the phenotype of 172 strains with the a−7g mutation, and only 56 of these were resistant to ethionamide (31). This could be due to inconsistencies in drug susceptibility testing or because the level of resistance conferred lay close to the breakpoint used in susceptibility testing but suggests that not all mutations within the *ethA-ethR* intergenic region result in levels of resistance similar to those that we observed for strains with the t−11c mutation (30).

Few studies have used quantitative drug susceptibility testing to correlate the ethionamide MIC with the genotype (32), so it is unclear to what extent individual mutations contribute to resistance and how they might interact. Although there may be additional mechanisms of ethionamide resistance that have yet to be identified, our results suggest that the t−11c mutation causes a modest increase in the ethionamide MIC but in combination with a mutation in the *inhA* promoter (considered to cause low-level ethionamide resistance [24]) leads to high-level resistance. Among the other ethionamide-resistant strains assayed, most had more than one mutation potentially contributing to their increased MIC. The pathway to clinical ethionamide resistance may therefore be the stepwise accumulation of multiple mutations rather than the selection of a single high-level-resistance-conferring mutation, as seen with some other antituberculosis drugs.

In the panel of clinical isolates that we selected to evaluate the phenotype associated with the t−11c mutation, we identified polymorphisms in other genes implicated in ethionamide resistance. We found four mutations at three positions in the *inhA* promoter region, all of which have been previously described (33). One of these strains had a t−8g *inhA* promoter mutation in combination with a nonsynonymous mutation (V18A) in *ndh*, which encodes a type II NADH dehydrogenase. Mutations in *ndh* can result in increased levels of NADH and reduce the level of binding of the isoniazid and ethionamide NAD adducts to their target, InhA (33). However, this strain had a low MIC, suggesting that neither of these mutations causes high-level resistance.

We cannot rule out the possibility of the existence of other ethionamide resistance-conferring mechanisms in our strains. EthA is 1 of 30 other monooxygenases within the M. tuberculosis genome (24), and a recently characterized monooxygenase, *mymA* (*Rv3083*) (27), was proposed to be an additional enzyme responsible for the activation of ethionamide. We identified strains with a 2,891-bp deletion spanning *mymA*, *lipR*, and half of *Rv3085* (Fig. S1). Two of these strains had no other known mutations associated with ethionamide resistance but were susceptible to ethionamide when a standard MIC cutoff was employed (Table 2), suggesting that *mymA* is not important for drug resistance in clinical isolates.

Our initial comparative transcriptional analysis identified only a limited number of genes whose level of expression was statistically significantly different from that in the control. This may have been due to the increased variability associated with the propagation of clinical isolates in culture media. We therefore looked at genes whose expression was highly divergent in the resistant strains in all pairwise comparisons. In addition to *ethA*, this identified *mazF5* and *mazE5*, which encode a toxin-antitoxin system, one of nine MazEF homologues in M. tuberculosis. A *mazF3*, *mazF6*, and *mazF9* triple-null mutant was less able to survive exposure to antituberculosis drugs (34), so these systems could potentially be involved in mediating resistance, although it is unclear how the downregulation of *mazE5* would influence drug susceptibility. Two tRNAs, *leuX* and *thrU*, were among the genes most highly upregulated in the resistant strains. Beyond a fundamental role in translation, tRNAs and their degradation products have been shown to regulate stress responses and adaptive changes in translation (35). It is therefore conceivable that the upregulation of these two tRNAs may be a manifestation of more global regulatory changes that have occurred during the evolution of drug resistance. Future studies comprising strains from different outbreaks and lineages are, however, needed to determine whether these transcriptional changes are limited to the XDR outbreak from KwaZulu-Natal in 2005 (1).

The treatment of MDR-TB is currently undergoing a revolution, with the introduction of new drugs and regimens (36). WHO has recently approved the use of a 9-month short course of therapy, and the 4-month intensive phase of this regimen includes ethionamide (or its analogue, prothionamide). Although the contribution of individual drugs to treatment efficacy is unclear, it is recommended that short-course treatment be withheld from MDR-TB patients with preexisting resistance to any individual drug. Pretreatment screening for ethionamide resistance is therefore critical for the implementation of short-course MDR treatment. However, testing for phenotypic susceptibility to ethionamide is notoriously difficult (37). Our results contribute to the development of a genetics-based resistance test, but further studies are required to define the interaction of diverse mutations and drug resistance-conferring loci as well as establish a clinically relevant critical concentration for ethionamide.

## MATERIALS AND METHODS

### Strains and growth conditions.

Three XDR isolates and 1 fully drug-susceptible clinical isolate from the LAM4 (KZN) spoligotype of M. tuberculosis (Table 1) were obtained from archived cultures from single colonies whose genomes had previously been sequenced (1). Cultures were grown in triplicate at 37°C in BD Difco Middlebrook 7H9 broth supplemented with BBL Middlebrook oleic acid-albumin-dextrose-catalase enrichment medium, 0.5% glycerol, and 0.01% Tween 80 with continuous shaking at 200 rpm. Additional strains were selected from the same collection on the basis of specific *ethA*, *ethR*, *inhA*, and *mymA* genotypes (Table 2) (1).

### RNA extraction and quality control. (i) RNA extraction.

RNA was harvested from 25-ml cultures grown to an optical density at 600 nm (OD_600_) of between 0.5 and 0.8, using a modified TRIzol method (38). Briefly, the cultures were centrifuged at 4,000 rpm for 20 min at 25°C and the pellet was resuspended in 1 ml of TRIzol reagent (Invitrogen, USA). Thereafter, approximately 100 μl of 0.1-mm zirconia/silica glass beads (BioSpec Products, USA) was added and the cultures were subjected to four pulses of bead beating, using a Roche MagNA Lyser instrument, at 7,000 rpm for 60 s with 2-min intermittent incubations on ice. Immediately after bead beating, 200 μl of chloroform was added, followed by centrifugation at 15,000 rpm for 15 min at 4°C and separation of the aqueous phase. The RNA was precipitated with 500 μl of 100% isopropanol and incubated at −20°C for 1 h. After centrifugation at 15,000 rpm for 10 min at 4°C, the RNA pellet was washed with 1 ml 75% ethanol, centrifuged at 10,000 rpm for 5 min at 4°C, and air dried. The RNA pellet was then dissolved in 30 μl of RNase-free water.

### (ii) DNase treatment and purification.

The RNA was subjected to DNase treatment using a DNase I RNase-free kit (Thermo Scientific, USA) per the manufacturer's instructions. The RNA was then purified using an RNeasy minikit (Qiagen, Germany), during which a second round of DNase digestion utilizing the RNase-free DNase set (Qiagen, Germany) took place. The integrity of the RNA samples was confirmed using a 23S rRNA/16S rRNA ratio (≥1.2) determined by an Experion StdSens analysis kit (Bio-Rad, USA).

### RNA sequencing and bioinformatics analysis. (i) RNA-seq library preparation.

A Qubit RNA assay kit (Invitrogen, USA) was used with a Qubit (version 2.0) fluorometer to quantify the RNA. Following RNA quantification, the rRNA was depleted using a Ribo-Zero magnetic kit (Illumina, USA). Enriched mRNA was analyzed on an RNA-specific E-gel EX 2% agarose gel (Invitrogen, USA) to confirm rRNA removal. After purification of the mRNA using an RNeasy minikit (Qiagen, Germany), RNA sequencing libraries were constructed using a NEBNext Ultra directional RNA library preparation kit for Illumina (New England BioLabs Inc., USA). The prepared libraries were indexed with NEBNext multiplex oligonucleotides for Illumina (New England BioLabs Inc. USA) and sequenced with 50-bp single-end reads on an Illumina HiSeq 2000 platform at the Norwegian Sequencing Centre, Oslo, Norway.

### (ii) Bioinformatics.

The sequence reads (Bioproject PRJNA414397, SRA SRP120003) were aligned to the M. tuberculosis H37Rv genome (NCBI accession number NC_000962.2) using the SeqMan NGen program from DNAStar Lasergene (version 11) software. Transcripts for each sample were quantified and normalized as the number of reads per kilobase per million reads (RPKM). The three replicate RPKM values for each sample were standardized on the basis of their mean transcript values and were used to assess gene expression and fold change differences in expression between isolates using the ArrayStar program (DNAStar). Pairwise comparisons between strains were conducted, with confidence intervals and statistics being determined using Student's *t* test and with multiple-testing corrections being made using the Benjamini-Hochberg correction to reduce the false discovery rate (FDR). Intergenic SNPs present only in the three XDR strains and not in the drug-susceptible strain were identified from whole-genome sequencing data from a previous study (1). A transcriptomic-genomic analysis was then conducted to identify promoter SNPs associated with at least a 4-fold up- or downregulation of the downstream gene.

### Whole-genome phylogeny.

Sequence reads for the four KZN strains were downloaded from the Sequence Read Archive (run accession numbers SRR832991, SRR833024, SRR833121, and SRR924700). The reads were aligned to the H37Rv genome (NCBI accession number NC_000962.3) using the SeqMan NGen program (DNAStar), resulting in median alignment depths ranging from 184 to 330 times for individual isolates. SNPs were called and filtered as previously described (39). The concatenated SNPs were used to create a distance-based neighbor-joining tree.

### RT-qPCR.

RNA was reverse transcribed into cDNA using an iScript cDNA synthesis kit (Bio-Rad, USA). Quantitative real-time PCR was conducted using iTaq Universal SYBR green supermix (Bio-Rad, USA) and forward and reverse primers for selected genes of interest. Primers were designed for *ethA*, *inhA*, *ethR*, and a housekeeping gene, *sigA* (see Table S5 in the supplemental material). Expression levels were normalized to the expression level of the reference gene, *sigA*.

### Flow cytometry promoter reporter assay.

To create a dual-color reporter, the Multisite Gateway three-fragment vector construction method (Invitrogen, USA) was used. The *ethA-ethR* intergenic region, mycobacterial codon-optimized Emerald GFP, and mycobacterial codon-optimized TagRFP constitutively expressed by the promoter pUV15 were individually cloned into entry vectors. These were combined with a destination vector based on an episomal mycobacterial vector containing a kanamycin resistance cassette (*aph*), the mycobacterial origin of replication, and the Escherichia coli origin of replication. Four separate *ethA-ethR* intergenic regions corresponding to the wild-type and mutant sequences (generated by PCR using genomic DNA from resistant clinical isolates) upstream of *ethA* and the same pair in the reverse orientation corresponding to the sequences upstream of *ethR* were used (Table S6). Additional plasmids with the *inhA* promoter with and without a g−17t mutation and a nonpromoter region (intragenic *katG* sequence) cloned in front of the GFP were constructed (Fig. S2). The promoter sequences for each construct were confirmed. The respective plasmids were transformed into H37Rv using standard protocols (40).

Strains harboring the dual-color reporters were grown to mid-log phase (OD_600_, 0.5 to 0.8) in 7H9 medium containing 25 mg/liter kanamycin. One milliliter of each strain was then filtered through a 10-μm-pore-size filter and acquired on a BD FACSAria III flow cytometer using BD DIVA software. A total of 100,000 events were recorded, with single-cell acquisition set at a threshold rate of ∼5,000 to 7,000 events per second. Green and red fluorescence were detected using the fluorescein isothiocyanate (FITC) and propidium iodide (PI) filters, respectively. The gating strategy employed during acquisition and software analysis, in which FlowJo (version 10) software was used, differentiated single cells/events on the basis of the relationship between cell size (forward scatter [FSC]) and granularity (side scatter [SSC]). Secondary gating on events with a red fluorescent signal was done using FlowJo software to ensure that only cells containing expression vectors were included in our analysis. The median fluorescent intensity (MFI) of the red and green fluorescent signals was extracted. The MFI of green fluorescence was normalized to the MFI of red fluorescence for each replicate before calculation of the median and standard deviation. A two-sided *t* test was used to determine statistical significance.

### Drug susceptibility testing.

One hundred microliters of three dilutions of each strain including 1 ×10^6^, 1 ×10^4^, and 1 ×10^3^ cells was plated onto quadrant plates containing BD Difco Middlebrook 7H10 agar with various concentrations of ethionamide (1.25, 2.5, 5, 10, 20, 40, and 80 mg/liter), and the number of CFU was counted after 3 weeks of incubation at 37°C.

### Global distribution of *ethA* promoter mutations.

A global data set of 5,310 M. tuberculosis strains from five continents (28) was searched for all instances of *ethA* promoter mutations. To identify individual mutation events arising across the phylogeny, we performed parsimony-based analysis using PAUP software, version 4.0b10 (29), as described by Manson et al. (28).

## Supplementary Material

Supplemental material
