# Supplementary material for: Whole-Transcriptome and -Genome Analysis of Extensively Drug-Resistant Mycobacterium tuberculosis Clinical Isolates Identifies Downregulation of ethA as a Mechanism of Ethionamide Resistance
Source: Antimicrob Agents Chemother. 2017 Nov 22;61(12):e01461-17. doi: 10.1128/AAC.01461-17 (PMC5700317; doi:10.1128/AAC.01461-17)
Supplement: Supplemental material [file supp_61_12_e01461-17__index.html]

Supplemental material 

# Whole-Transcriptome and -Genome Analysis of Extensively Drug-Resistant Mycobacterium tuberculosis Clinical Isolates Identifies Downregulation of *ethA* as a Mechanism of Ethionamide Resistance

## Supplemental material

- Supplemental file 1 -

  Table S1

  XLSX, 560K
- Supplemental file 2 -

  Table S2

  XLSX, 10K
- Supplemental file 3 -

  Table S3

  XLSX, 11K
- Supplemental file 4 -

  Table S4

  XLSX, 13K
- Supplemental file 5 -

  Table S5

  XLSX, 9.9K
- Supplemental file 6 -

  Table S6

  XLSX, 11K
- Supplemental file 7 -

  Fig. S1 and S2

  PDF, 526K
