# Supplementary material for: Whole-Transcriptome and -Genome Analysis of Extensively Drug-Resistant Mycobacterium tuberculosis Clinical Isolates Identifies Downregulation of ethA as a Mechanism of Ethionamide Resistance
Source: Antimicrob Agents Chemother. 2017 Nov 22;61(12):e01461-17. doi: 10.1128/AAC.01461-17 (PMC5700317; doi:10.1128/AAC.01461-17)
Supplement: Supplemental material [file AAC.01461-17_zac012176746s7.pdf]

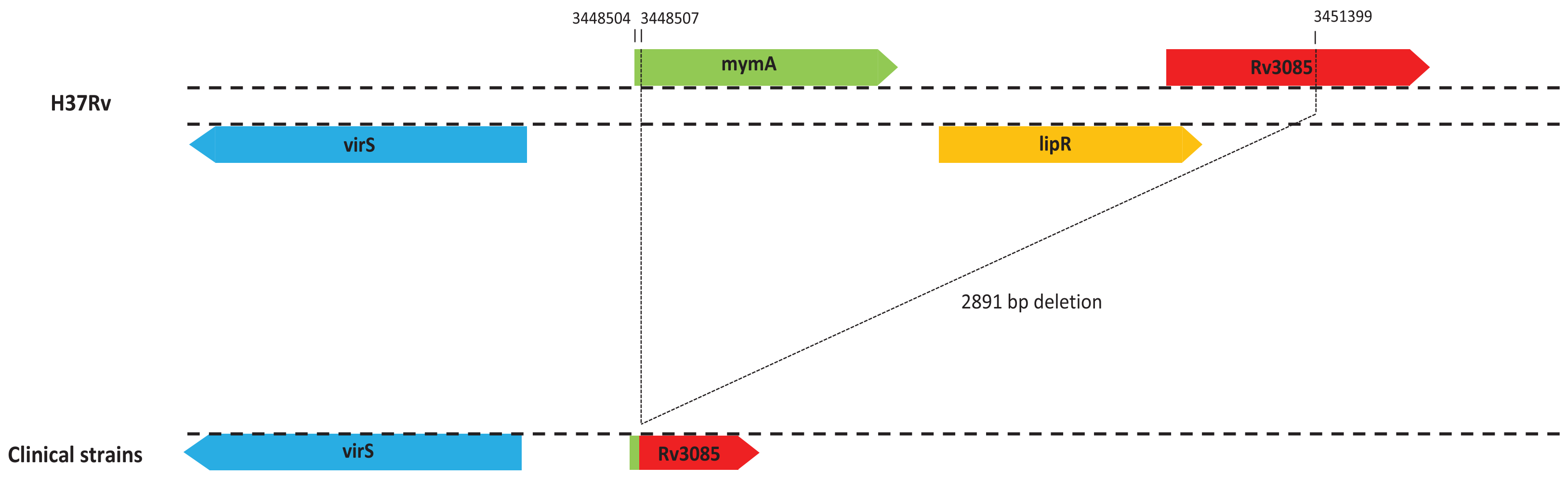

FIG S1 Schematic representation of the 2891 bp deletion detected and sequenced confirmed in our clinical strains. The deletion spans across genes mymA, lipR and half of Rv3085

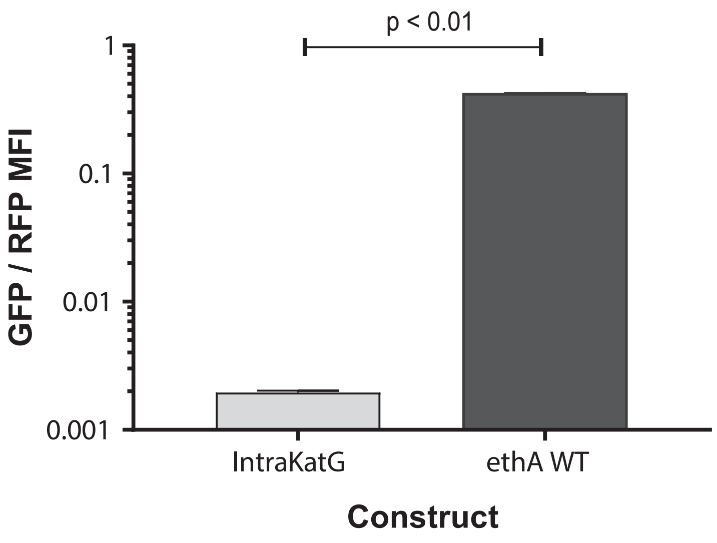

FIG S2 Anaylsis of promoter activity between a promoterless negative control (IntraKatG) and ethA WT. MFI represents mean fluorescent intensity of green fluorescent protein (GFP) normalized to red fluorescent protein (RFP). Results are representative of two replicates for each construct run in the same experiment. Statistical significance is indicated on the bar chart
